# A Flexible Wearable Sensor Based on Laser-Induced Graphene for High-Precision Fine Motion Capture for Pilots

**DOI:** 10.3390/s24041349

**Published:** 2024-02-19

**Authors:** Xiaoqing Xing, Yao Zou, Mian Zhong, Shichen Li, Hongyun Fan, Xia Lei, Juhang Yin, Jiaqing Shen, Xinyi Liu, Man Xu, Yong Jiang, Tao Tang, Yu Qian, Chao Zhou

**Affiliations:** 1 Institute of Electronic and Electrical Engineering, Civil Aviation Flight University of China, Deyang 618307, China; xingxiaoqing@cafuc.edu.cn (X.X.);; 2 Key Laboratory of Flight Techniques and Flight Safety, CAAC, Deyang 618307, China; 3 School of Mathematics and Physics, Southwest University of Science and Technology, Mianyang 621010, China; 4 College of Electronic and Information, Southwest Minzu University, Chengdu 610225, China; 5 School of Flight Technology, Civil Aviation Flight University of China, Deyang 618307, China

**Keywords:** laser-induced graphene, laser energy accumulation, morphology, flexible wearable sensor, motion posture capture

## Abstract

There has been a significant shift in research focus in recent years toward laser-induced graphene (LIG), which is a high-performance material with immense potential for use in energy storage, ultrahydrophobic water applications, and electronic devices. In particular, LIG has demonstrated considerable potential in the field of high-precision human motion posture capture using flexible sensing materials. In this study, we investigated the surface morphology evolution and performance of LIG formed by varying the laser energy accumulation times. Further, to capture human motion posture, we evaluated the performance of highly accurate flexible wearable sensors based on LIG. The experimental results showed that the sensors prepared using LIG exhibited exceptional flexibility and mechanical performance when the laser energy accumulation was optimized three times. They exhibited remarkable attributes, such as high sensitivity (~41.4), a low detection limit (0.05%), a rapid time response (response time of ~150 ms; relaxation time of ~100 ms), and excellent response stability even after 2000 s at a strain of 1.0% or 8.0%. These findings unequivocally show that flexible wearable sensors based on LIG have significant potential for capturing human motion posture, wrist pulse rates, and eye blinking patterns. Moreover, the sensors can capture various physiological signals for pilots to provide real-time capturing.

## 1. Introduction

Graphene is a two-dimensional (2D) material known for its excellent electrical conductivity, thermal conductivity, and mechanical strength [1,2]. It has extensive applications in energy storage, flexible sensors, and electronic devices, offering broad application prospects across various industries [3,4]. However, high manufacturing costs and inefficient production methods currently hinder its large-scale industrialization [5,6]. Unlike traditional processes, the fabrication of laser-induced graphene (LIG) is a novel preparation approach [7]. LIG can be directly formed in air, which simplifies and expedites the fabrication process while allowing for customized laser patterns through computer programming [8,9]. This maskless preparation method involves low costs and exhibits high efficiency, showcasing tremendous potential for sustainable development and the large-scale industrial production of graphene [10,11].

Understanding the formation mechanism of LIG to optimize its preparation, characteristics, and applications is crucial. The primary factors influencing the LIG formation mechanism are carbon precursor materials and types of lasers [11,12,13]. Carbon precursor materials typically comprise high-molecular-weight polymers containing aromatic compounds or natural substances, such as lignin. Upon laser irradiation, aromatic compound-containing materials, such as polyimide (PI), polyetherimide (PEI), and poly ether ether ketone (PEEK), experience localized high-temperature and high-pressure conditions [14,15,16,17], resulting in the direct disruption of their C–O, C=O, and C–N bonds [18]. Consequently, gas molecules, including those of N and O, are released while the C atoms recombine to form LIG [19]. Heat treatment can also be performed on materials containing lignin, such as pine, coconut shell, potato peel, and other natural materials, to convert them into LIG as they contain abundant aromatic subunits [20,21,22]. Various factors influence LIG formation during laser processing, and different laser types or changes in laser parameters distinctly affect the morphology and performance of LIG [23]. Different wavelength lasers, such as a 355 nm ultraviolet femtosecond laser [24], a 522 nm green femtosecond laser [25], and a 10.6 μm CO_2_ infrared laser [16], can be used to irradiate the surface of various carbon precursor materials to produce LIG. However, different laser parameters, including the pulse duration, radiation energy, scanning speed, and laser power, significantly affect the morphology and performance of LIG [26,27,28,29,30,31]. The effects of these laser parameters on LIG characteristics can be studied by varying individual parameters [23]. In the most current studies on LIG preparation, a 10.6 μm CO_2_ infrared laser is commonly used to irradiate PI surfaces to investigate the influence of parameters, such as radiation energy, laser power, and scanning speed, on LIG formation. However, few studies have specifically focused on the surface morphology evolution and performance of LIG modified by the laser energy accumulation by varying the laser scanning time.

Based on our group’s previous research [32], we investigated the effect of altering the laser scanning time on LIG characteristics while keeping the other laser parameters constant. The LIG was observed to transition from a surface porous structure to a relatively flat structure with fewer holes and an abundant porous interior on the surface with an increasing laser scanning time. This unique porous structure enhanced the extensive applications of LIG. In this study, we successfully utilized LIG to assemble a high-precision flexible wearable sensor for pilots’ motion posture capture. The LIG-based flexible wearable strain sensor, with limited relevant applications, aims to be innovatively utilized to monitor physiological parameters during training sessions in civil aviation pilot simulators. In this study, the application of the LIG-based flexible sensor in monitoring the training process of civil aviation pilots is a first-time endeavor. It is expected to serve as a valuable resource for capturing and analyzing the motions involved in pilot aviation flight operation.

## 2. Experimental Process

### 2.1. Materials and Preparation Procedure

**Figure 1:**
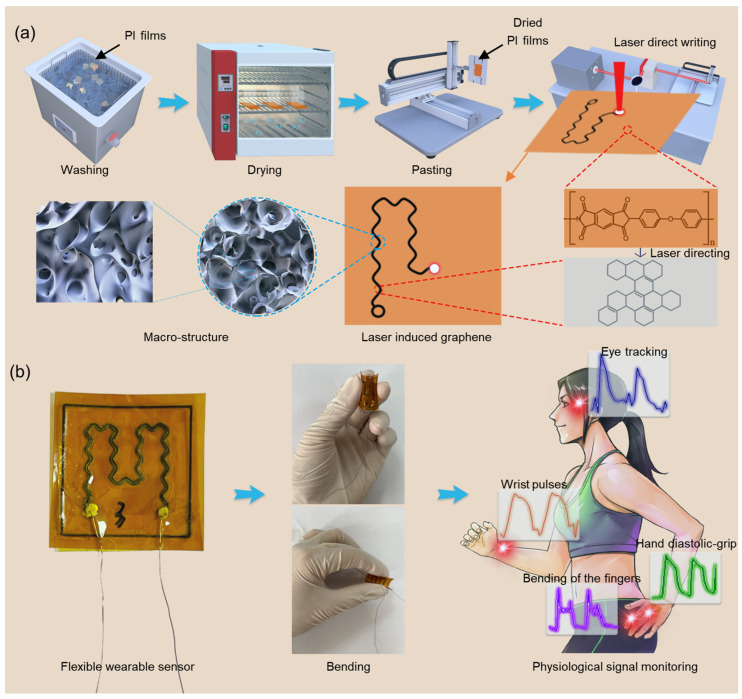
Overview of laser-induced graphene (LIG) devices and related applications. (**a**) Illustration of the fabrication process of LIG films. (**b**) Assembly and application of LIG.

The laser-induced graphene preparation process is shown in Figure 1. In Figure 1a, PI films (Shenzhen Jihongda Plastic Products Co., Ltd., Shenzhen, China) with a size of 2.5 mm × 2.5 mm were cleaned by using deionized water in an ultrasonic cleaner (Chun Rain Inc., Shenzhen, China), and then removed and dried using a blow drying oven (DHG-202, Shaoxing Subo Instrument Co. Ltd., Shaoxing, China) at 50 °C for 2 h. It was fixed on a three-dimensional moving stage using PET (Wing Tai Co., Zhongshan, China) tape and then scanned using a CO_2_ infrared laser (Synrad P150, Novanta Corporation, Bedford, MA, USA) with a laser power of 12.4 W, a pulse repetition frequency of 20 kHz, and a scanning speed of 105 mm/s, so that the graphene was scanned at the scanning path position on the PI surface. A serpentine-shaped LIG pattern with a scan size of 1.5 mm × 1.5 mm was generated. And after several scans, the shape of the LIG surface was generated with a porous and microporous shape. As shown in Figure 1b, the generated LIG can be prepared into flexible wearable sensors and encapsulated with PI tape with a 50 μm thickness (Shenzhen Jihongda Plastic Products Co., Shenzhen, China). These sensors can be bent into various shapes and can also be directly attached to the skin surface using PET tape to monitor the body posture, especially the pulse as well as finger movement condition for capturing.

### 2.2. Material Characterization

In this experiment, focused ion beam scanning electron microscopy (SEM; Thermo Scientific Helios 5 CX, Thermo Fisher Scientific Inc., Waltham, MA, USA) was employed to observe the surface morphology of LIG. An energy-dispersive X-ray spectroscopy (EDS; Thermo Scientific Helios 5 CX, USA) analysis was performed to detect the types and distribution of elements in LIG. The positions of the PI and LIG peaks were detected by X-ray diffraction (XRD; Rigaku Ultima IV, Rigaku Corporation, Akishima, Japan). Fourier-transform infrared spectroscopy (FTIR; Thermo field Nicolet iS5, Thermo Fisher Scientific Inc., Waltham, MA, USA) was employed to detect the functional group types and differences between LIG and PI. The positions of the LIG peaks were detected using a Renishaw inVia confocal microRaman spectrometer (Raman, Gloucestershire, UK). X-ray photoelectron spectroscopy (XPS; FEI ESCALAB Xi+, Thermo Fisher Scientific Inc., Waltham, MA, USA) was employed to detect the atomic contents of LIG and PI substrates. Atomic force microscopy (AFM; NT-MDT Spectrum Instruments, Moscow, Russia) was employed to observe the morphology and roughness of LIG. A real-time resistance test was conducted using a digital multimeter (RIGOL DM3058E, RIGOL Technologies Co., Ltd., Suzhou, China), and a tensile tester (WNMC, Beijing, China) was used for the tensile strain test.

## 3. Results and Discussion

### 3.1. Surface Morphology Characterization

**Figure 2:**
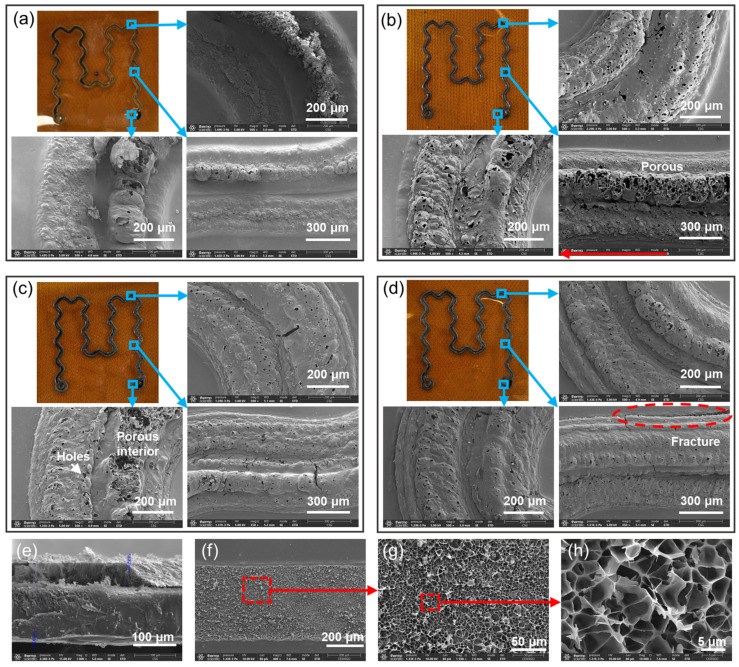
Influence of the scanning account on the microscope of the LIG at corners, curves, and circles, respectively. (**a**) Scanning conducted one time. (**b**) Scanning conducted two times. (**c**) Scanning conducted three times. (**d**) Scanning conducted four times. (**e**) Cross–sectional SEM image of LIG with three scans. (**f**–**h**) SEM images of the PI surface after the detachment of LIG with three scans at different scales.

The LIG sample and its SEM surface images after one to four laser scans are shown in Figure 2. The initial observations revealed that pale yellow traces appeared on the PI surface with a single laser scan, accompanied by the sporadic generation of discontinuous black material. With an increase in the number of laser scans from two to four, gradual and continuous LIG formation was observed. During laser scanning, localized temperature elevation induced by the laser energy triggered thermal decomposition reactions. Insufficient laser energy led to slight breaking in the molecular structure of PI, resulting in pale yellow traces [33]. However, when the laser energy was supplied, a large amount of heat energy was released, leading to significant fracture of the PI molecules and the production of gases and small-molecule products. Some of these products may have recombined to form black coke or LIG. As shown in Figure 2a, there are pale yellow traces and black material on the PI surface in the SEM images at different locations. LIG-like substances were formed with numerous unruptured bubbles present in the sample.

The process of continuous LIG generation is shown in Figure 2b, where the SEM images reveal multiple surface holes and unruptured bubbles with remarkable clarity. These holes were formed due to the thermal decomposition of PI induced by laser energy, generating bubbles and small-molecule products. Subsequently, these products underwent pore structure formation due to thermal expansion, effectively enhancing the surface area of LIG. The presence of unruptured bubbles indicates incomplete thermal decomposition caused by insufficient laser energy. Figure 2c shows the LIG sample after three scans, which exhibits a smoother surface. However, beneath this smoother LIG surface, there was an abundance of three-dimensional pore structures [34]. This phenomenon can be attributed to the accumulation of laser energy on the PI surface during multiple scans, resulting in enhanced thermal decomposition and volatilization that generated gases and produced more LIG build-up on the surface. With an increasing number of scans, laser energy accumulated within the PI material, resulting in more PI molecules undergoing thermal decomposition. The aggregation effect in PI confined certain gases and small-molecule products within localized regions, internally forming a structure with abundant pores while maintaining the LIG surface’s sleek external appearance. Figure 2d shows the SEM image of LIG produced after four consecutive laser scans, exhibiting a smooth surface. Nevertheless, evidence suggests fracture occurrence in the red region in the lower right corner of the figure. This fracture can potentially be ascribed to enhanced energy accumulation resulting from repeated laser scans, causing localized sections within LIG to become harder and more brittle.

The cross-sectional SEM image shown in Figure 2e demonstrates the dense and impermeable nature of the material at the PI position, while revealing a porous structure in the LIG region. Subsequent SEM tests conducted at various scales on the PI surface after detaching LIG consistently confirm its porous structure (Figure 2f–h).

This discovery substantiates the explanation provided for Figure 2c: under multiple scanning cycles, PI undergoes thorough reactions, and the generated LIG fills the surface pores. Due to the reduction in surface pores, the generated gas cannot be released. Meanwhile, an abundance of porous structures forms internally within the LIG, resulting in a characteristic where the surface porosity of the LIG is low, while its internal structure is rich in pores.

### 3.2. Spectroscopy Results

**Figure 3:**
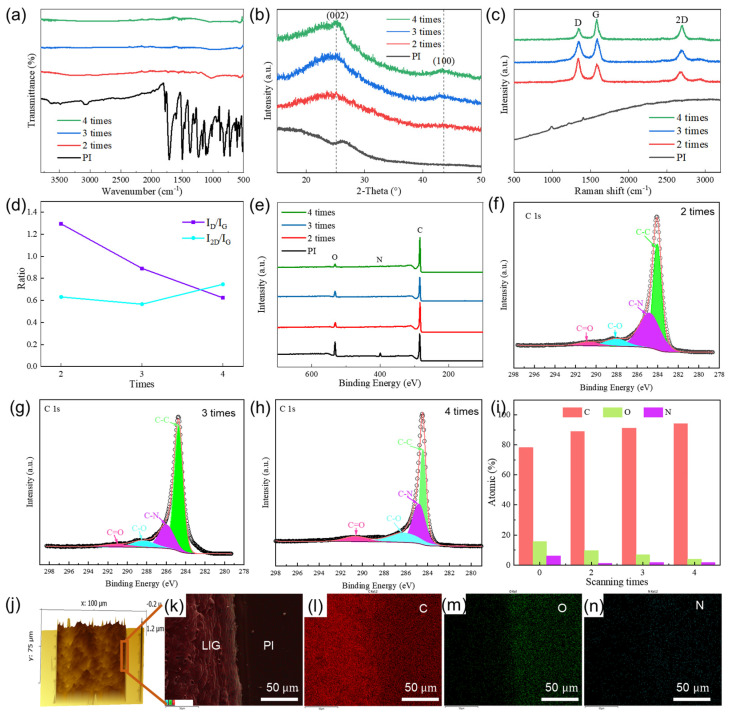
Characterization of LIG powders prepared using different laser scanning times. (**a**) FTIR spectroscopy. (**b**) X–ray diffraction patterns. (**c**) Raman spectra. (**d**) Raman spectra peak analysis. (**e**) XPS patterns and atomic content ratios of C, O, and N. (**f**–**h**) Peak deconvolution and fitting of C1s XPS spectra with three scans. (**i**) C, N, and O elemental content analysis. (**j**) AFM of LIG prepared at a number of three scanning times. (**k**–**n**) The SEM-EDS mappings of LIG and PI, including carbon, oxygen, and nitrogen, respectively.

The LIG powder data provided more accurate characterization results. However, one laser scan could not produce LIG and collect the powder, so we characterized the LIG powder and carbon precursor PI with two to four laser scans. The spectral detection results of the LIG and PI with two to four laser scans are shown in Figure 3. As shown in Figure 3a, the PI and LIG functional groups are easily detected by FTIR spectroscopy. Evidently, the absorption peak diminished during the conversion of PI to LIG. Within the wavenumber range of 500–1800 cm^−1^, there was a noticeable decrease in the intensity of the stretching vibration peaks of the C–N bond, C=O bond, and aromatic ring. In addition, within the wavenumber range of 2800–3000 cm^−1^, there was attenuation in the peak intensity of the C–H bond’s stretching vibration. Figure 3b shows the XRD patterns of the LIG and PI obtained using different laser scanning times with utmost precision. Compared with the pattern of PI, that of the LIG exhibited distinct (002) and (100) peaks [33]. The presence of these peaks reflects unique characteristics that are specific to the layer arrangement pattern of graphene materials, which appear at smaller (2θ = 25.18°) and larger angles (2θ = 43.6°). Figure 3c shows the Raman spectra of the PI and LIG. The LIG exhibited three distinct peaks that were not observed for PI, and the positions of the LIG peaks remained consistent across different laser scanning times. In particular, the D peak was observed at 1343.2 cm^−1^, the G peak at 1580.1 cm^−1^, and the 2D peak at 2700.1 cm^−1^ [35]. These findings provide evidence for the graphene composition of LIG. The intensity of the D peak reflects defects in the LIG, with higher intensities indicating larger defects. Meanwhile, the G peak corresponds to the lattice vibration of the LIG, with stronger intensities indicating enhanced graphene lattice integrity. Further, the 2D peak represents vibrations between two carbon atoms, and large values suggest fewer LIG layers and a higher overall material performance. Variations were observed in the D, G, and 2D peak intensities for different scanning times. Accordingly, the spectra shown in Figure 3c were analyzed. A lower I_D_/I_G_ ratio corresponds to a reduced number of defects, a higher I_2D_/I_G_ ratio, and fewer LIG layers. The calculation of the PI as the carbon precursor was not performed. Figure 3d shows that as the scanning times increased, the I_D_/I_G_ values were 1.29, 0.89, and 0.62, and the I_2D_/I_G_ values were 0.63, 0.57, and 0.75. Raman detection revealed that there were the least LIG defects for four scanning times. Meanwhile, the LIG defects for two scanning times were comparatively more prevalent.

The XPS spectrum of the LIG is shown in Figure 3e. Figure 3f–h shows the XPS analysis and C1s peak fitting for the LIG with 2–4 scanning cycles. The results of the analysis indicate that the LIG exhibits excellent quality. The PI contained higher proportions of C, O, and N atoms, and with an increasing number of scans, more C atoms were incorporated, while the contents of O and N atoms gradually decreased. An analysis indicated that this phenomenon can be attributed to the recombination of C atoms during the preparation process, along with the formation of gases from N and O atoms. Figure 3f shows that the C atom contents for different scanning times were 78.22%, 89.07%, 91.26%, and 94.23%, whereas those for the O atom were 15.61%, 9.74%, 6.94%, and 3.99%. Similarly, the N atom contents were 6.17%, 1.19%, 1.8%, and 1.78%. The products under different scanning times generally contained C atoms, along with a minimal O content, which indicates that LIG was being formed rather than other substances, such as graphene oxide.

The results presented in Figure 3c–f demonstrate the exceptional quality of the LIG formed after four laser scanning events. However, it is evident from the SEM image in Figure 2d that the LIG prepared using four laser scans was highly susceptible to fracture. Consequently, our next step was to analyze the LIG formed after three scanning events to enhance its adhesion and flexibility. Figure 3g shows the AFM result of the LIG formed after three scanning events, revealing a surface characterized by undulations but relatively flat with a roughness value of only 0.066 μm. Furthermore, Figure 3h–k present the EDS analysis results for LIG formed after three scanning events, revealing brighter coloration for C elements compared to O and N elements on the PI substrate surface. The number of surface C atoms in LIG exceeded that observed in PI, while there were lower O and N atom concentrations compared to those observed in PI.

### 3.3. Sensing Performance of Flexible Sensors

**Figure 4:**
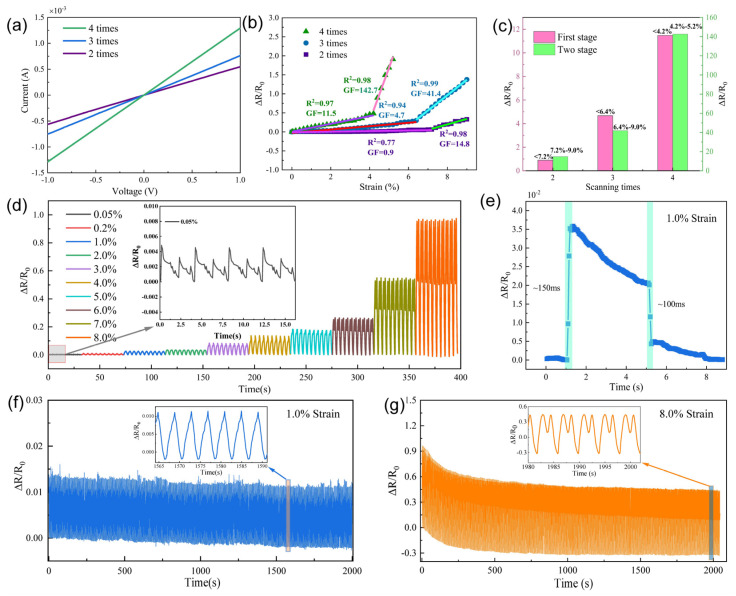
Performance test of LIG. (**a**) I–V testing of flexible sensors with two to four scans. (**b**) Sensitivity testing of flexible sensors with two to four scans stomach scans and two-stage linear fitting. (**c**) GF for different stretch lengths. (**d**) The stability test of the flexible sensor with three scanning times was performed for 10 cycles with tensile strain rates of 0.05%, 0.2%, 1.0%, 2.0%, 3.0%, 4.0%, 5.0%, 6.0%, 7.0%, and 8.0%, respectively. (**e**) The time response of a flexible sensor with three scanning times was tested at a tensile strain of 1.0%. (**f**) Stability tests lasting 2000 s were performed on a flexible sensor with three scanning times at a tensile strain of 1.0%. (**g**) Stability tests lasting 2000 s were performed on a flexible sensor with three scans at a tensile strain of 8.0%.

Using a CO_2_ infrared laser to directly write on the PI surface, we achieved a remarkable single-step in situ formation of LIG with excellent electrical conductivity. This also led to the fabrication of a flexible sensor with superior mechanical properties. The performance evaluation and analysis results of the flexible sensor are presented in Figure 4. However, it is worth noting that the electrical conductivity of LIG with only one scan was insufficient. The sensor maintains a stable and original conductive pathway with constant resistance in the absence of strain. However, when subjected to strain, certain conductive pathways may undergo fracture, while new pathways are formed. As the applied strain increases, the number of fractured conductive pathways grows, while the formation of new pathways gradually decreases, leading to an increased relative change in resistance observed by the sensor. When the sensor is released from a stretched state, it gradually reverts to the original conductive pathway, resulting in a gradual reduction in relative resistance change until it reaches zero. This mechanism enables the sensor to sensitively respond to strain and recover to its original state after release.

Hence, we are currently investigating flexible sensors fabricated using two to four scans. Figure 4a shows the current–voltage test results of these flexible sensors, revealing their outstanding resistance stability. Figure 4b shows the test results of the relative resistance changes observed in the three flexible sensors. Sensors fabricated using varying scanning times exhibited distinct characteristics during the stretch–release test. Using a two-stage formula, we successfully achieved linear fitting to establish a relationship between the tensile strain and relative resistance change. Notably, an expression exists for sensitivity (*GF*) in this relationship, which encompasses relative resistance change and tensile strain change.

(1) $$GF=\frac{\delta (R-R_{0})/R_{0}}{\delta \varepsilon },$$

where R represents real-time resistance, R_0_ represents initial resistance, and ε represents tensile strain.

As shown in Figure 4b, the sensor fabricated using two scans exhibited a sensitivity of only 0.9 at a stretched length in the range of 0.0% to 7.2% and released, which increased to 14.8 within the range of 7.2% to 9.0%. Conversely, the sensor fabricated using three scans demonstrated a sensitivity of 4.7 within the range of 0.0% to 6.4%, significantly improving to 41.4 within the range of 6.4% to 9.0%. The flexible strain sensor exhibited outstanding performance, as illustrated in Table 1. This sensor demonstrated superior sensitivity compared to the sensors utilizing alternative conductive materials and fabrication processes [36,37,38,39,40,41,42,43,44]. The sensor fabricated using four scans exhibited even higher sensitivities, reaching up to 11.5 in the range of 0.0% to 4.2%, and as high as 142.7 in the range of 4.2% to 9.0%, which is more visually represented in Figure 4c. This indicates a more pronounced change in relative resistance compared with sensors of the first two categories. However, it is worth noting from Figure 2 that the LIG’s surface was susceptible to breaking. In cases where there was no replacement or packaging using more flexible materials for the substrate, employing only the PI substrate led to potential damage [45]. Furthermore, during sensitivity testing, a limited tensile strain range was observed for this flexible sensor. Therefore, all of our later performance evaluations will be investigated using flexible sensors fabricated in a triple laser scanning fashion.

The stability test results of the flexible sensors under various tensile strains are shown in Figure 4d. Tests were conducted using tensile strains of 0.05%, 0.2%, 1.0%, 2.0%, 3.0%, 4.0%, 5.0%, 6.0%, 7.0%, and 8.0% for ten cycles; the results revealed the outstanding stabilities of the flexible sensors. In addition, a small strain test was conducted on a flexible sensor, and the flexible sensors were tested for low detection limits (detection limit: 0.05%). The time response and relaxation test results for the flexible sensor are shown in Figure 4e. The response time was ~150 ms, while the relaxation time was ~100 ms. Finally, as shown in Figure 4f, the flexible sensor underwent endurance tests for 2000 s with 1.0% tensile strain and 8.0% tensile strain, as shown in Figure 4g. In both conditions, excellent stability was observed.

### 3.4. Applications

**Figure 5:**
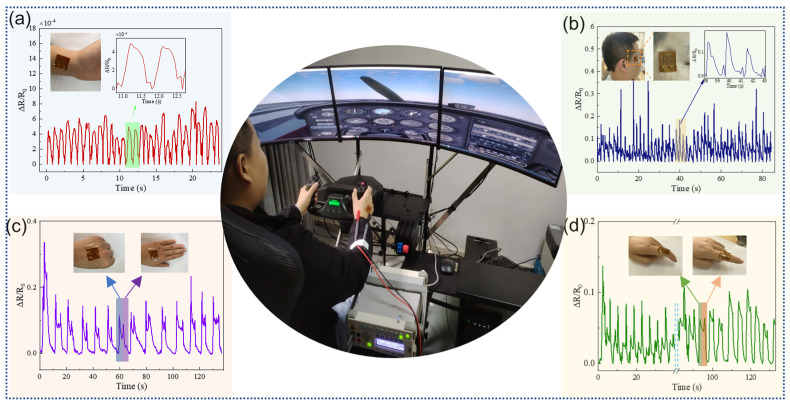
Flexible strain sensor based on LIG for body detection and motion capture for pilots. (**a**) Pulse detection. (**b**) Blink detection. (**c**) Hand grip and stretch test. (**d**) Finger curvature detection.

As an emerging sensor technology, flexible strain sensors have demonstrated significant potential for the high-sensitivity detection of minute physiological signals [34,46]. By attaching a flexible strain sensor to the human wrist, the real-time capture of the pulse vibration frequency can be performed [47]. This is crucial for early disease detection, health status monitoring, and heart rate variability analysis. Flexible strain sensors overcome the limitations of traditional electrocardiography (ECG) and blood pressure measurements by providing a more convenient, accurate, and comfortable method for heart monitoring, particularly for health trackers and patients with cardiovascular diseases. In this study, we attached a flexible strain sensor to the wrist position because of its excellent flexibility and fit. This effectively reduced external interference and error. Figure 5a shows the results obtained by examining wrist pulses, which demonstrated the superiority of the flexible strain sensor in detecting small physiological signals. Overall, the sensors offer an efficient, accurate, and convenient means for human physiological monitoring.

A flexible strain sensor, affixed to the corner of the eye, enabled the precise capture of minute changes in the eye movement without direct contact with the ocular surface. It avoided extrusion or interference with the eye, making it highly valuable for assessing eye health and studying eye movement control [48]. As shown in Figure 5b, the flexible strain sensor was placed at the corner of the eye to detect variations in relative resistance caused by different blink forces, providing a useful reference point. When attached to either the back of the hand or finger, this sensor captured finger flexibility and gesture control, which are considerably important for rehabilitation training, sports performance evaluation, and advancement in gesture recognition technology [49,50]. An accurate analysis of finger strain patterns facilitates the effective capture of finger movements and postures while enhancing rehabilitation therapy and human–computer interaction technology. Figure 5c,d show the excellent stability exhibited by this sensor during the relaxation of the back of the hand and bending of the fingers.

## 4. Conclusions

Through characterization and performance tests on the prepared flexible sensors, the comprehensive evaluation revealed that LIG and the flexible sensors fabricated using accumulative laser energy three times exhibited superior performance without altering the PI substrate. They possessed a high sensitivity of 41.4 and exceptional stability at tensile strains of 0.2%, 1.0%, 2.0%, 3.0%, 4.0%, 5.0%, 6.0%, 7.0%, and 8.0%. In addition, the sensors exhibited a low detection limit (0.05%), rapid response time (~150 ms), rapid relaxation time (~100 ms), and good stability under 1.0% tensile strain (2000 s) and 8.0% tensile strain (2000 s). Furthermore, it could be used to test small physiological signals, such as the pulse rate, blinking of the eyes, relaxation of the hand, and finger bending motions. We verified that multiple laser scans gradually flatten the surface of LIG while creating an interior that is rich in pores or holes, providing valuable insights into its application potential. The specific surface area of the porous structure was increased, enabling sensitivities toward minute changes, thereby accurately capturing subtle variations in human motion posture and facilitating high-precision motion capturing for pilots. Furthermore, the flat surface morphology remained stable even after numerous stretches and bends, which is crucial for motion capturing studies over extended periods. This application of LIG-based sensors not only yields valuable insights into pilots’ physiological responses and manual actions during training but also expands the applicability of flexible sensors to diverse fields. Apart from its advantages for highly precise human motion posture monitoring, which are high sensitivity, high flexibility, and a rapid response time, it can also be used in capacitors to enhance charge storage and release, as adsorption materials or filters, and as flexible biosensors or gas sensors.

## Figures and Tables

**Table 1:** Performance comparison of flexible strain sensors with various materials.

Processes	Conducting Materials	Flexible Substrate	Sensing Range (Strain%)	Gauge Factor (GF)	References
Interface ablation	LIG	PI	0–2%	24.80	[36]
Laser directing	LIG	PDMS	0–20%	15.79	[37]
Laser directing	rGO	PDMS	0–140%	12.12	[38]
/	Thick graphene platelets (GnPs)	Epoxy	0–0.67%	33.00	[39]
Screen printing technique	Graphene nanoplatelets (GNPs)/Inks	Coated fabrics	0–5%	30.00	[40]
/	CNTs	Acrylic substrate	0–150%	26.70	[41]
Transfer printing	AgNWs	PDMS	0–100%	2.87	[42]
/	PVA-CT-Ag-Al-Gly (PCAAG)	Hydrogel	0–350%	2.10	[43]
/	CMC/PAA/Fe^3+^/LiCl	Hydrogel	0–400%	6.19	[44]
Laser directing	LIG	PI	0–9%	41.40	This work

## Data Availability

Data is contained within the article.
